# Variation in the caprine keratin-associated protein 15-1 (KAP15-1) gene affects cashmere fibre diameter

**DOI:** 10.5194/aab-62-125-2019

**Published:** 2019-03-26

**Authors:** Mengli Zhao, Huitong Zhou, Jon G. H. Hickford, Hua Gong, Jiqing Wang, Jiang Hu, Xiu Liu, Shaobin Li, Zhiyun Hao, Yuzhu Luo

**Affiliations:** 1 Gansu Key Laboratory of Herbivorous Animal Biotechnology, Faculty of Animal Science and Technology, Gansu Agricultural University, Lanzhou 730070, China; 2 International Wool Research Institute, Faculty of Animal Science and Technology, Gansu Agricultural University, Lanzhou 730070, China; 3 Gene-Marker Laboratory, Faculty of Agriculture and Life Sciences, Lincoln University, Lincoln 7647, New Zealand

## Abstract

Keratin-associated proteins (KAPs) are a structural component of cashmere fibre, and variation in some KAP genes (*KRTAP*s) has been associated with a number of caprine fibre traits. In this study, we report the identification of *KRTAP15-1* in goats. Sequence variation in the gene was detected using the polymerase chain reaction single-strand conformation polymorphism (PCR-SSCP) technique in 250 Longdong goats, and six variants (named A to F) containing eight single nucleotide polymorphisms (SNPs) were identified. Five of the SNPs were non-synonymous and would lead to putative amino acid changes. Reverse-transcription polymerase chain reaction (RT-PCR) analysis revealed that *KRTAP15-1* was expressed in secondary hair follicles but not in heart tissue, liver tissue, lung tissue, kidney tissue or the longissimus dorsi muscle. Despite being rich in cysteine, the caprine KAP15-1 protein possesses a high content of serine and moderate content of glycine and phenylalanine. Association analyses revealed that *KRTAP15-1* variant A was associated with decreased mean fibre diameter (MFD), and this effect appeared to be dominant; while variant C was found to be associated with increased MFD, the effect being recessive. The findings suggest that caprine *KRTAP15-1 *is highly polymorphic and that variation in this gene affects cashmere MFD.

## 1 Introduction

Hair and cashmere are produced by the primary and the secondary hair follicles, respectively, of cashmere goats. As a consequence of the characteristic of being softer, lighter and stronger, with better insulating properties, cashmere fibres are sometimes called “soft gold”, this reflecting how highly the fibre is valued. The price of cashmere fibres is comparatively much higher than wool and mohair, and cashmere fleece weight, mean fibre diameter (MFD) and unstraightened fibre length are the most important traits that determine the economic return for the production of cashmere.

The main components of cashmere fibres are keratins (Ks), which form keratin intermediate filaments and keratin-associated proteins (KAPs), which form a matrix cross-linking the keratin intermediate filaments. The Ks and KAPs are therefore believed to play an important role in determining the characteristics of the fibre.

KAPs have a high content of either cysteine or glycine and tyrosine, and based on this content, the proteins can be divided into three broad groups: the high-sulfur (HS) group, which contains less than 30 mol % cysteine; the ultra-high-sulfur (UHS) group, which contains more than 30 mol % cysteine, and the high glycine and tyrosine (HGT) group, which has 35 mol %–60 mol % glycine and tyrosine (Gong et al., 2016). Within these groups, the KAPs can be further subdivided into families based on their sequence similarity, and over 100 KAP genes (called *KRTAP*s) belonging to 27 families have been identified to date across mammalian species (Gong et al., 2010).

KAP15-1 is a single gene-member family belonging to the HS-KAP group. The KAP15-1 gene (*KRTAP15-1*) has been described in mice and humans (Pruett et al., 2004; Rogers et al., 2002) and recently in sheep (Li et al., 2018). In sheep, variation in *KRTAP15-1* has been reported to affect wool yield (Li et al., 2018). Despite a caprine *KRTAP15-1* sequence having been deposited in the NCBI GenBank database (accession number AY510116.1), little is known about *KRTAP15-1* variation and its effect on fibre traits in this species.

In this study, we attempted to identify *KRTAP15-1* in goats, to search for potential variation in the gene and to investigate its effect on cashmere fleece traits.

## 2 Materials and methods

### 2.1 Goats and DNA samples

Two hundred and fifty-three Longdong cashmere goats fed at the Yu sheng Cashmere Goat Breeding Company in Huan County of Gansu Province were investigated. At 1 year of age, cashmere fibres were collected by combing the goats, and the weight of fibre collected per goat was measured. A sample of fibre from the mid-side region was collected from each goat and sent for the measurement of mean crimped fibre length and MFD at the Inner Mongolia Agricultural University, Inner Mongolia, China.

Blood samples from these goats were collected directly onto FTA cards (Whatman BioScience, Middlesex, UK). A two-step washing procedure was used to purify the goat genomic DNA for polymerase chain reaction (PCR) amplification from 1.2 mm punches of the dried blood spots, using the protocol described in Zhou et al. (2006).

### 2.2 Animal tissues

Three 3-year-old Longdong cashmere goats were slaughtered, and tissue from the skin, heart, liver, lungs, kidneys and longissimus dorsi muscle was collected and rapidly frozen for storage in liquid nitrogen. The separation of primary and secondary hair follicles from the skin tissue used the method described by Jin et al. (2011). Briefly, the skin sample was cut into a strip of a width of 1.0 cm. Next, the hair follicle bulbs were exposed by removing the subcutaneous fat using a dissecting needle and the follicle tissues separated from the surrounding tissues. Once isolated, the hair follicles were sorted under a microscope into primary and secondary follicles, this being based on whether they had sweat glands or not.

### 2.3 Polymerase chain reaction amplification

A comparison of the caprine *KRTAP15-1* sequence (accession number AY510116.1) and the ovine *KRTAP15-1* sequences (accession numbers MH742372 – MH742375) suggested that the primers designed for ovine *KRTAP15-1* (Li et al., 2018), would also amplify a 495 bp fragment covering the entire coding sequence of caprine *KRTAP15-1*. The sequences of these primers were 5'-GAACTCAGAACTCCCAACAG-3' and 5'-TAACCATGAGGTGACTGGAG-3', and they were synthesised by Sangon Biotech Co., Ltd (Shanghai, China).

Amplifications were undertaken in Bio-Rad S1000 thermal cyclers (Bio-Rad, Hercules, CA, USA) and performed in a 20 µL reaction including the purified genomic DNA from one 1.2 mm punch of dried blood, 0.25 µM of each primer, 2.0 µL of 10× PCR buffer (supplied with the DNA polymerase enzyme), deoxyribonucleoside triphosphates (dNTPs) at 150 µM (Takara, Dalian, China), 2.5 mM Mg${}^{2+}$ and 0.5 U of Taq DNA polymerase (Takara), with deionised water (${\mathrm{dH}}_{2}O$) to make up the volume. The PCR amplification conditions consisted of an initial denaturation at 94 ${}^{\circ }$C for 5 min, followed by 35 cycles of 94 ${}^{\circ }$C denaturation for 30 s, 60 ${}^{\circ }$C annealing for 30 s and 72 ${}^{\circ }$C extension for 30 s, and a final extension at 72 ${}^{\circ }$C of 5 min. The quality of PCR products was examined using agarose gel electrophoresis (1  % gel in 1× TBE (Tris–borate–EDTA) buffer).

### 2.4 Single-strand conformation polymorphism (SSCP) analysis of amplicons

A 1.0 µL aliquot of the PCR amplicons was mixed with 8.0 µL of loading dye (98 % formamide, 10 mM ethylenediaminetetraacetic acid (EDTA), 0.025 % bromophenol blue, 0.025 % xylene cyanol). Samples were denatured at 95 ${}^{\circ }$C for 10 min and then quickly cooled in wet ice before being loaded onto 16 cm × 18 cm, 12 % acrylamide : bisacrylamide (37.5:1) (Bio-Rad) gels. Electrophoresis was carried out at in 0.5× TBE buffer at 230 V and 11.5 ${}^{\circ }$C for 22.5 h. The gels were silver-stained according to the method of Byun et al. (2009).

### 2.5 Sequencing of *KRTAP15-1* variants and sequence analyses

Amplicons that appeared to be homozygous upon analysis of the SSCP gels were sequenced in both directions by the Beijing Genomics Institute (Beijing, China). Variants that appeared to be present in a heterozygous form were sequenced using an approach described previously (Gong et al., 2011). Briefly, a band corresponding to the variant was excised as a gel slice from the polyacrylamide gel, macerated, and then used as a template for re-amplification with the original primers. The second amplicon was then sequenced directly.

The alignment of DNA sequences, translation, comparisons and the construction of the phylogenetic tree were undertaken using DNAMAN version 5.2.10 (Lynnon BioSoft, Vaudreuil, Canada).

The coding sequence of a caprine *KRTAP15-1* sequence (accession number AY510116.1) was used to BLAST search the Caprine Genome Assembly (ASM170441 v1) to determine its chromosomal location.

### 2.6 Reverse-transcription polymerase chain reaction

Trizol reagent (Invitrogen, Carlsbad, CA, USA) was used to extract total RNA from between 50 and 100 mg of each of the five tissue samples and the secondary follicles. The quality and concentration of the RNA extracted was checked using 2 % agarose gels electrophoresis and UV spectrophotometry, respectively.

The PrimeScript^™^ RT Reagent Kit with gDNA Eraser (Perfect Real Time, Takara) was used to produce cDNA, following the manufacturer's instructions. This cDNA was amplified using a second set of PCR primers (5'-ATCTTCCGCAGTCCCTG-3' and 5'-GATGACCGGCAACTCCT-3') located within the *KRTAP15-1* coding region, which would amplify a 161 bp fragment. This amplification used the conditions and thermal profile described above, but the genomic DNA template was replaced with a 0.8 µL aliquot of the cDNA. The caprine β-actin gene was selected as an internal reference standard with the primers 5'-AGCCTTCCTTCCTGGGCATGGA-3' and 5'-GGACAGCACCGTGTTGGCGTAGA-3'. PCR products were examined by electrophoresis in 1.0 % agarose gels.

### 2.7 Statistical analyses

All statistical analyses were undertaken using SPSS v24.0 (IBM, NY, USA). General linear mixed-effects models (GLMMs) were used to evaluate the effect of the absence or presence (coded as 0 or 1, respectively) of *KRTAP15-1* variants on the three cashmere traits that had been measured.

For those variants with sufficiently common homozygous forms (>1 % of all genotypes), a second set of analyses was performed with the number of variant copies present included (in place of absence or presence), followed by planned orthogonal contrasts to ascertain whether additive, dominant or recessive effects were present. These models were conducted in an identical manner to the GLMMs used for testing the presence/absence of each variant. As with the absence or presence models, each variant was tested in separate models and then subsequently included in a model with all the variants that met the criteria (i.e. sufficient homozygosity) present. A Bonferroni correction was applied to reduce the probability of false positive results during the multiple comparisons in these models.

Gender and sire were found to affect (P<0.05) all the fibre traits and so they were included in the models as fixed and random factors, respectively. Birth rank was not found to affect fibre characteristics and thus was not included in the models. Only the main effects were tested. Unless indicated, all P values were considered statistically significant when P<0.05. Trends were noted when 0.05≤P<0.1.

## 3 Results

### 3.1 Chromosome location of caprine *KRTAP15-1*

**Figure 1:**
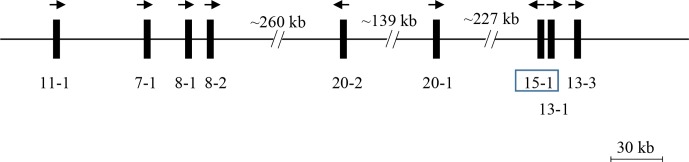
KAP genes identified on goat chromosome 1. The newly identified *KRTAP15-1* is shown in the box, along with eight previously identified *KRTAP*s. Vertical bars represent *KRTAP*s, with the names of the genes being indicated below (i.e. 11-1 represents *KRTAP11-1*). The arrows represent the direction of transcription. The distances between these genes are approximate and refer to the caprine genome assembly NC_030808.1.

A BLAST search of the Caprine Genome Assembly ASM170441 v1 using a caprine *KRTAP15-1* coding sequence (accession number AY510116.1) revealed a 411 bp open reading frame (ORF) with 99 % sequence identity on caprine chromosome 1 (NC_030808.1: nt3853204_nt3853614) in a region harbouring eight previously identified caprine *KRTAP*s including *KRTAP11-1*, *KRTAP7-1*, *KRTAP8-1*, *KRTAP8-2*, *KRTAP20-2*, *KRTAP20-1*, *KRTAP13-1* and *KRTAP13-3* (Wang et al., 2018) (Fig. 1). This ORF was approximately 227.4 kb downstream of *KRTAP20-1* and approximately 3.3 kb and 15.0 kb upstream of *KRTAP13-1* and *KRTAP13-3*, respectively, but it had a different transcriptional direction to these nearby *KRTAP*s.

### 3.2 Sequence variation in caprine *KRTAP15-1*

**Figure 2:**
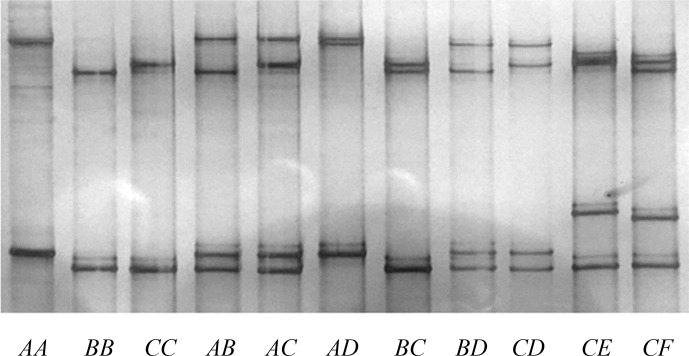
PCR-SSCP of caprine *KRTAP15-1*. Six unique SSCP banding patterns, corresponding to six different variant sequences (A to F) are shown in either homozygous or heterozygous forms.

**Figure 3:**
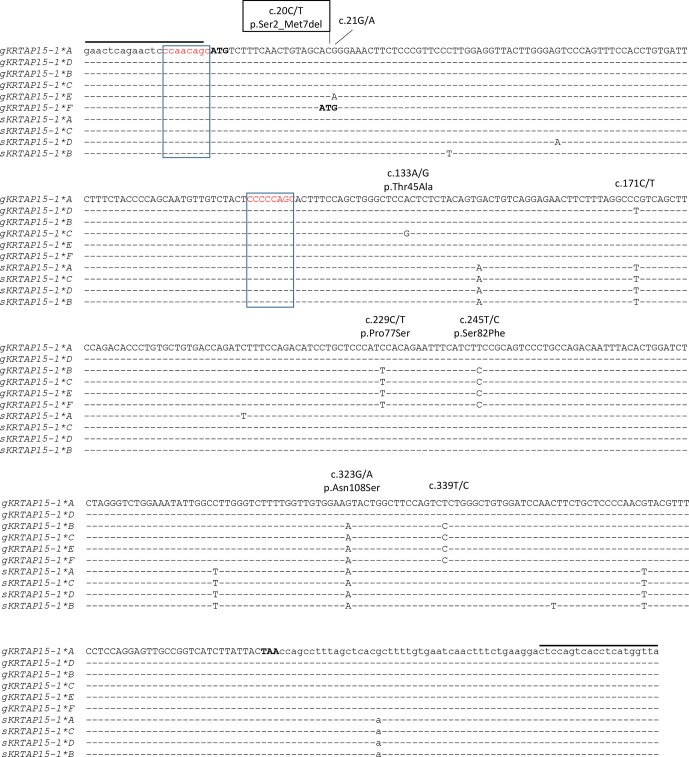
Alignment of the *KRTAP15-1* variants identified in goats and sheep. Eight single nucleotide polymorphisms (SNPs) identified in caprine *KRTAP15-1* are marked above the sequences, with the non-synonymous SNPs being indicated with the putative amino acid changes and the SNP that would result in an alternative start codon being boxed. Dashes signify homology with the caprine KRTAP15-1*A sequence, with the coding sequence being shown in upper case and the non-coding sequences being shown in lower case. The notional ATG start codon and TAA stop codon are shown in bold. Two putative chi-like sequences are boxed. The primer-binding regions are indicated with horizontal lines. The goat sequences are indicated with a prefix “g”, while the sheep sequences are indicated with a prefix “s”. Numbering of nucleotide and amino acid positions follows the guidelines of the Human Genome Variation Society (HGVS) nomenclature (http://varnomen.hgvs.org/, last access: 21 February 2018). The GenBank accession numbers of these sequences are MH742372 (sKRTAP15-1*A), MH742373 (sKRTAP15-1*B), MH742374 (sKRTAP15-1*C) and MH742375 (sKRTAP15-1*D).

Six unique banding patterns (A to F) were identified in the Longdong cashmere goats by PCR-SSCP analysis (Fig. 2). Either one or a combination of two banding patterns were observed for each goat, and this is consistent with it being either homozygous or heterozygous for variation in the gene. Sequencing of PCR amplicons representative of these different banding patterns revealed six unique nucleotide sequences and eight single nucleotide polymorphisms (SNPs). Five of these SNPs were non-synonymous, and one non-synonymous SNP would lead to the creation of an alternative start codon at the seventh codon position (Fig. 3). This would result in a loss of six N-terminal amino acids in the protein. Four of the SNPs were found to be in linkage, and these were c.229C/T, c.245T/C, c.323G/A and c.339T/C, with the haplotypes through these SNPs being either T-C-A-C or C-T-G-T.

In the 250 Longdong cashmere goats investigated, the frequency of the six *KRTAP15-1* variants A, B, C, D, E and F was 41.6 %, 27.5 %, 27.3 %, 3.2 %, 0.2 % and 0.2 %, respectively. The common variants were A, B and C, while the common genotypes were AA, BB, CC, AB, AC and BC, these collectively accounting for 92.8 % of the goats. The other genotypes were rare and occurred at a frequency of less than 5 %.

### 3.3 Phylogenetic relationship of caprine *KRTAP15-1* to other HS-*KRTAP*s

**Figure 4:**
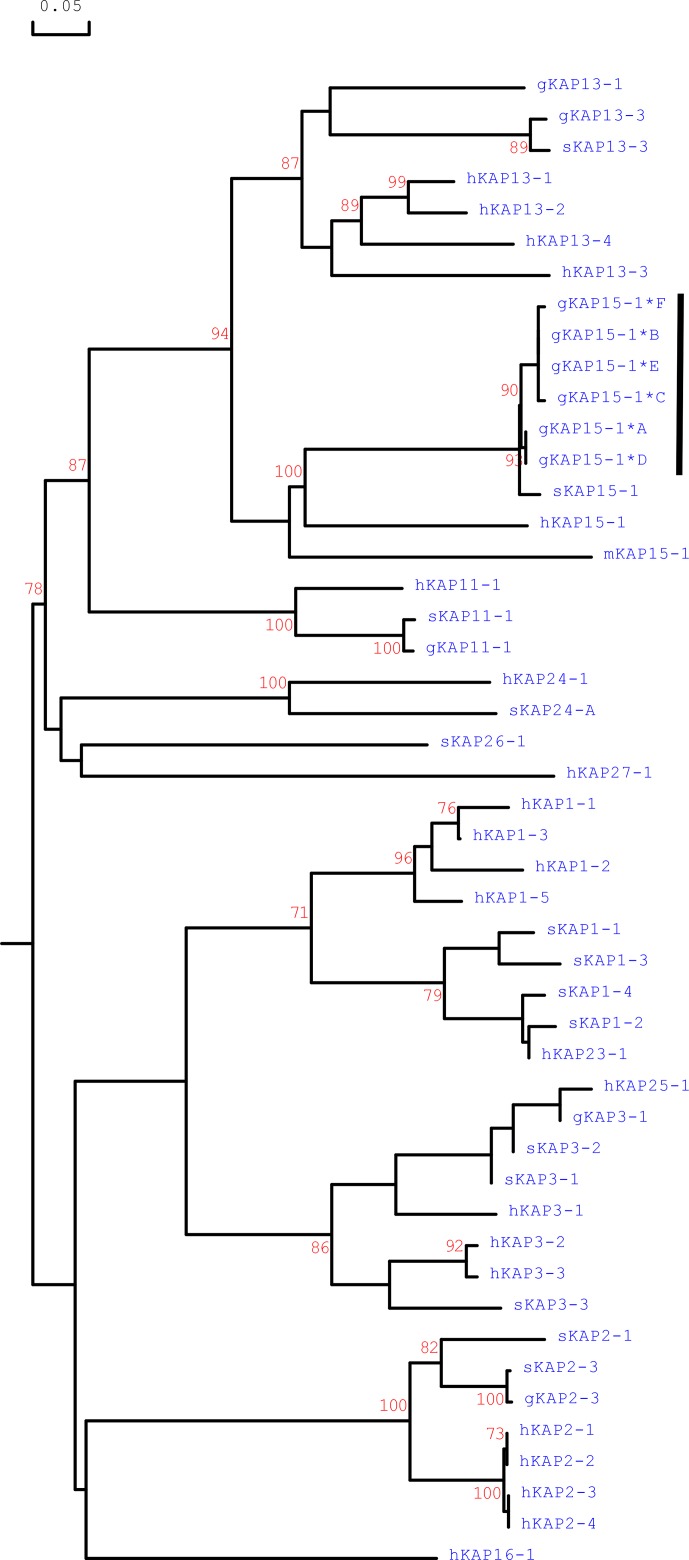
Phylogenetic tree of HS-KAPs from goats, sheep, humans and mice. The tree was constructed using the amino acid sequences or the predicted amino acid sequences. The caprine KAPs are indicated with a prefix “g”, while the sequences of sheep, humans and mice are indicated with prefix “s”, “h” and “m”, respectively. The numbers at the forks indicate the bootstrap confidence values and only those equal to or higher than 70 % are shown. The newly identified caprine KAP15-1 sequences are indicated with a horizontal bar, and the GenBank accession numbers for other HS-KAPs are NM_001285774 (gKAP3-1), NM_001285767.1 (gKAP11-1), AY510115 (gKAP13-1), JX426138 (gKAP13-3), X01610 (sKAP1-1 and sKAP1-4), HQ897975 (sKAP1-2), NM_001159761 (sKAP1-3), P02443 (sKAP2-1), U60024 (sKAP2-3), M21099 (sKAP3-1), M21100 (sKAP3-2), M21103 (sKAP3-3), HQ595352 (sKAP11-1), JN377429 (sKAP13-3), MH742372 (sKAP15-1), JX112014 (sKAP24-1), KX644903 (sKAP26-1), NM_030967.2 (hKAP1-1), NM_030966.1 (hKAP1-3), NM_001257305.1 (hKAP1-4), NM_031957.1 (hKAP1-5), NM_001123387.1 (hKAP2-1), NM_033032.2 (hKAP2-2), NM_001165252.1 (hKAP2-3), NM_033184.3 (hKAP2-4), NM_031958.1 (hKAP3-1), NM_031959.2 (hKAP3-2), NM_033185.2 (hKAP3-3), NM_175858.2 (hKAP11-1), NM_181599.2 (hKAP13-1), NM_181621.3 (hKAP13-2), NM_181622.1 (hKAP13-3), NM_181600.1 (hKAP13-4), NM_181623.1 (hKAP15-1), NM_001146182.1 (hKAP16-1), NM_181624.1 (hKAP23-1), NM_001085455.2 (hKAP24-1), NM_001128598.1 (hKAP25-1), NM_203405.1 (hKAP26-1), NM_001077711.1 (hKAP27-1) and NM_013713 (mKAP15-1).

Phylogenetic analysis of the caprine *KRTAP15-1* sequences with all of the HS-*KRTAP*s identified in goats and sheep and the *KRTAP15-1* sequences from humans and mice revealed that the caprine *KRTAP15-1* sequences were clustered with the *KRTAP15-1* sequences from sheep, human and mice and separated from ovine and caprine *KRTAP*s from other HS-KAP families (Fig. 4). This suggests the sequences identified represent caprine *KRTAP15-1*, and based on this analysis the variants A–F were formally named CAPHI-KRTAP15-1*A to CAPHI-KRTAP15-1*F to be consistent with the KAP/*KRTAP* nomenclature proposed by Gong et al. (2012).

### 3.4 Predicted amino acid composition of caprine KAP15-1

The putative caprine KAP15-1 gene is predicted to encode a protein of 136 amino acid residues. The most common amino acid is serine (20.6 mol %–21.3 mol %), followed by glycine (11.0 mol %), phenylalanine (8.8 mol %–9.6 mol %), cysteine (7.4 mol %), threonine (6.6 mol %–8.1 mol %), tyrosine (5.9 mol %), proline (5.2 mol %–5.9 mol %), asparagine (5.5 mol %–5.9 mol %), leucine (5.2 mol %), valine (5.2 mol %), glutamine (5.2 mol %) and arginine (4.4 mol %). Other amino acids are rare or absent in the protein.

### 3.5 Expression of caprine *KRTAP15-1*

**Figure 5:**
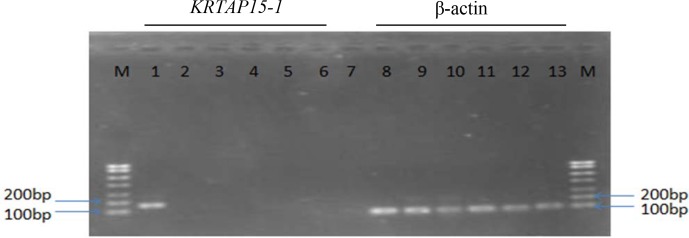
RT-PCR results for detection of β-actin and *KRTAP15-1* in six tissues derived from Longdong cashmere goats. M: 750 bp DNA marker; lanes 1 and 8: secondary hair follicle tissue; lanes 2 and 9: heart tissue; lanes 3 and 10: liver tissue; lanes 4 and 11: lung tissue; lanes 5 and 12: kidney tissue; lanes 6 and 13: longissimus dorsi muscle tissue; lane 7: negative control.

The concentration of RNA extracted from the secondary follicles and five tissues ranged from 931 to 1050 ng µL${}^{-1}$. Expression of *KRTAP15-1* was detected in secondary hair follicles from the Longdong goats but not in other tissues, including heart tissue, liver tissue, lung tissue, kidney tissue and tissue from the longissimus dorsi muscle (Fig. 5).

### 3.6 Association of *KRTAP15-1* variation with cashmere fibre traits

Of the six variants identified, variants D, E and F were rare (with a frequency of less than 5 %) in the Longdong cashmere goats investigated. These variants were removed from the association study, and analyses were only carried out for variants A, B and C. All of these variants also had sufficiently common homozygous forms to enable the copy number analyses (i.e. >1 % of all genotypes).

In the absence or presence models, the presence of variant A was found to be associated with decreased cashmere MFD (absent: 13.6±0.06 µm; present: 13.4±0.05 µm; P=0.006) (Table 1). No associations were detected for the other variants.

**Table 1:** Association of caprine *KRTAP15-1* variants with cashmere traits (mean ± SE).${}^{*}$

Trait (unit)	Variant	Absent			Present		P value
		Mean ± SE	n		Mean ± SE	n	
Cashmere yield (g)	A	417±6.0	74		418±4.8	157	0.861
	B	420±5.3	117		415±5.1	114	0.357
	C	419±5.1	119		416±5.2	112	0.509
Crimped fibre length (cm)	A	4.2±0.06	74		4.3±0.05	157	0.126
	B	4.3±0.06	117		4.3±0.06	114	0.487
	C	4.3±0.55	119		4.2±0.56	112	0.110
Mean fibre diameter (µm)	A	13.6±0.06	**74**		13.4±0.05	**157**	**0.006**
	B	13.4±0.05	117		13.4±0.05	114	0.997
	C	13.4±0.05	119		13.5±0.05	112	0.358

${}^{*}$ Estimated marginal means and standard errors (SEs) of those means from general linear mixed-effects models. “Gender” and “sire” were included in the models as a fixed factor and a random factor, respectively. P<0.05 is in bold.

When the number of variant copy was considered, goats that did not contain variant A produced fibres with higher mean fibre diameter than goats that contained one or two copies of A (Table 2). Goats that carried two copies of variant C produced fibre with higher MFD than goats that did not have C or that had one copy of C (Table 2).

**Table 2:** Association of caprine *KRTAP15-1* variant copy number with cashmere traits (mean ± SE).${}^{*}$

Trait (unit)	Variant	Absent			One copy			Two copies		P value
		Mean ± SE	n		Mean ± SE	n		Mean ± SE	n	
Cashmere yield (g)	A	417±6.0	74		417±5.1	116		423±7.7	41	0.721
	B	420±5.3	117		413±5.5	95		426±10.0	19	0.289
	C	420±5.1	119		415±5.5	93		422±10.2	19	0.622
Crimped fibre length (cm)	A	4.2±0.06	74		4.3±0.06	116		4.4±0.08	41	0.222
	B	4.3±0.06	117		4.3±0.06	95		4.3±0.11	19	0.606
	C	4.3±0.06	119		4.2±0.06	93		4.2±0.11	19	0.273
Mean fibre diameter (µm)	A	$13.6\pm 0.06^{a}$	74		$13.4\pm 0.05^{b}$	116		$13.4\pm 0.08^{b}$	41	0.022
	B	13.4±0.05	117		13.4±0.06	95		13.6±0.10	19	0.343
	C	$13.4\pm 0.05^{b}$	119		$13.4\pm 0.05^{b}$	93		$13.7\pm 0.10^{a}$	19	0.024

${}^{*}$ Estimated marginal means and standard errors (SEs) of those means derived from general linear mixed-effects models. “Gender” and “sire” were included in the models as a fixed factor and a random factor, respectively. Means within rows that do not share a superscript letter (${}^{a}$ or ${}^{b}$) are significantly (P<0.05) different and are given in bold.

## 4 Discussion

This study reports the identification of caprine *KRTAP15-1*, and describes its effect on cashmere fibre traits. The caprine *KRTAP15-1* gene was clustered with other HS-*KRTAP*s and HGT-*KRTAP*s in a region on chromosome one and in an arrangement similar to that found in sheep (Li et al., 2018). The gene was expressed in secondary fibre follicles in the skin, and it exhibited sequence variation. This variation appeared to affect cashmere MFD.

While the expression of *KRTAP15-1* was not investigated in the primary follicles, the detection of *KRTAP15-1* mRNA in the secondary follicles and apparent absence of expression in other tissues, including heart, liver, lung, kidney and longissimus dorsi muscle, suggests that *KRTAP15-1* is restricted to expression in the skin. This is consistent with the expression pattern detected for caprine *KRTAP20-1* (Wang et al., 2018) and caprine *KRTAP20-2* (Wang et al., 2017) but different to that reported for caprine *KRTAP11-1*. Jin et al. (2017) reported that this gene was strongly expressed in skin, heart and liver. The reason for this is currently unknown.

While KAP15-1 could be classified as belonging to the HS group (Rogers et al., 2006; Gong et al., 2012), the putative caprine KAP15-1 protein would contain a lower level of cysteine and a higher amount of serine than might be expected. This difference is not unprecedented, and it is similar to what has been observed with the HS-KAPs: KAP24-1 and KAP26-1 (Zhou et al., 2012; Li et al., 2017). The caprine KAP15-1 protein would also be rich in phenylalanine. Having a high level of phenylalanine has only been reported for the KAP15 family but has not been found in other HS KAPs (Li et al., 2018). The functional consequence of this is unknown, but it has been suggested that the possession of a high level of phenylalanine may facilitate interaction between KAP15-1 and the keratin intermediate filaments (KIFs) via a ring-stacking mechanism (Li et al., 2018).

Caprine *KRTAP15-1* appears to be polymorphic, with a high level of polymorphism being detected in a reasonably small group of cashmere goats. While ovine *KRTAP15-1* is polymorphic (Li et al., 2018), the level of polymorphism observed in sheep (four variants) was lower than detected here in the goats (six variants). Moreover, the number of SNPs identified in caprine *KRTAP15-1* is higher than that identified in the sheep homologue, with eight SNPs found in caprine *KRTAP15-1* compared to four SNPs in ovine *KRTAP15-1* (Li et al., 2018). The detection of eight SNPs in a 456 bp PCR fragment (excluding the primer binding regions) would give a density of approximately 17.5 SNPs per kilobase. This is much higher than the average SNP density of 4.9 SNPs per kilobase described for sheep (Kijas et al., 2009). Little is known about how this extensive variation came about in goats, but the linkage of SNPs suggests that gene conversion or non-reciprocal genetic exchange may have occurred and that this has contributed to the accumulation of sequence variation. The presence of two chi-like sequences (5'-CCAACAGC-3' from c.-8 to c.-1 and 5'-CCCCCAGC-3' from c.107 to c.114, reverse complementary to 5'-GCTGTTGG-3' and 5'-GCTGGGGG-3', respectively) in the caprine *KRTAP15-1* sequences supports this conclusion, and it appears to be similar to what has been reported for ovine *KRTAP1-n* (Rogers et al., 1994).

The association results obtained from the absence or presence models and the copy number models appear to match. For variant A, the absence of this variant was associated with a higher MFD than the presence of either one copy or two copies of the variant (see Table 2). This compares favourably with the single-variant absence or presence models, which reveal that the absence of variant A is associated with increased MFD, when compared to the presence of A (see Table 1). For variant C, goats that had one copy of this variant had no difference in MFD compared to goats that did not have variant C. This effect of variant C was not observed in the single-variant absence or presence models, but the finding might have been biased by the reasonably small number of CC goats investigated (n=19).

It is interesting to note that despite both variants A and C appearing to have an effect on MFD, the nature of effects observed for these two variants appear to be different. The models suggest that variant A has a dominant effect, while variant C has a recessive effect. The reason for this is unknown, and it may simply once again reflect bias from the smaller number of CC goats, but it could favour the production of finer cashmere fibres, should A be selected for.

The effect of *KRTAP15-1* in the cashmere goats appears to be different to that found in sheep. In sheep, *KRTAP15-1* is found to mainly affect wool yield, but it only had a trend of association with wool MFD (Li et al., 2018). Unfortunately fibre yield was not measured in the Longdong goats in this study. Whether this means that *KRTAP15-1* has a larger effect on MFD in cashmere fibres from goats than in wool from sheep cannot be easily resolved. It might be as a consequence of the fineness of the fibres, as cashmere fibres are much finer than wool fibres, with the average MFD of the cashmere samples investigated in this study being 13.3 µm versus 19.2 µm for the wool samples investigated in Li et al. (2018). A small change in MFD in the goats may be more pronounced and thus more easily reach significance.

When compared to the other *KRTAP*s in goats, *KRTAP15-1* appears to have a unique effect on cashmere traits. Of the 12 previously identified *KRTAP*s in goats, effects on cashmere fibre traits have been investigated for three: *KRTAP8-2* (Liu et al., 2007), *KRTAP20-1* (Wang et al., 2018) and *KRTAP20-2* (Wang et al., 2017). All of these *KRTAP*s are located on the same chromosome as *KRTAP15-1*. Variation in *KRTAP8-2* (Liu et al., 2007) was found to be associated with cashmere weight and MFD but not with fibre length, while variation in *KRTAP20-1* and *KRTAP20-2* was associated with cashmere weight and fibre length (Wang et al., 2017, 2018), and only a trend of association was observed with MFD with *KRTAP20-1* (Wang et al., 2018). Despite using the same population of cashmere goats as for the analysis of *KRTAP20-1* and *KRTAP20-2* (Wang et al., 2017, 2018), variation in *KRTAP15-1* is found to be associated with MFD but not with cashmere weight and fibre length. This suggests that different *KRTAP*s may have different effects on cashmere traits. In this context, the effect of *KRTAP15-1* on MFD would seem to be unlikely to be due to linkage to other *KRTAP*s on the same chromosome.

## Data Availability

The original data are available upon request to the corresponding author.
